# Identification of the transgene insertion site for an adipocyte-specific adiponectin-cre model and characterization of the functional consequences

**DOI:** 10.1080/21623945.2021.1880083

**Published:** 2021-02-10

**Authors:** Jared S. Farrar, Joseph C. Lownik, Grayson W. Way, Matthew C. Rodriguez, Francesco S. Celi, Rebecca K. Martin

**Affiliations:** a Center for Clinical and Translational Research, Virginia Commonwealth University School of Medicine, Richmond, VA, USA; b Department of Microbiology and Immunology, Virginia Commonwealth University School of Medicine, Richmond, VA, USA; c Department of Internal Medicine, Division of Endocrinology, Diabetes and Metabolism, Virginia Commonwealth University School of Medicine, Richmond, VA, USA

**Keywords:** Adipoq-cre, adipocyte, insertion site, passenger genes, bacterial artificial chromosome

## Abstract

The *Adipoq-Cre* transgenic mouse is widely used in the development of adipocyte-specific genetic manipulations for the study of obesity and type 2 diabetes. In the process of developing a new mouse model utilizing the adipocyte selective *Adipoq-Cre* transgenic mouse, strong genetic linkage between a gene of interest, *Adam10*, and the *Adipoq-Cre* transgene was discovered. Whole-genome sequencing of the *Adipoq-Cre* transgenic mouse model identified the genomic insertion site within the *Tbx18* gene locus on chromosome 9 and this insertion causes a significant decrease in *Tbx18* gene expression in adipose tissue. Insertion of genes *Kng2, Kng1, Eif4a2* and *Rfc4* also occurred in the *Adipoq-Cre* transgenic mouse, and these passenger genes may have functional consequences in various tissues.

## 1. Introduction

Few tools have proven as powerful as the *Cre* recombinase for the investigation of gene function in mouse models. Both spatial and temporal genetic manipulations can be performed using cell-type-specific promoters to drive the expression of *Cre* recombinase in combination with *Cre* recognition (loxP) sites in a gene, or genes, of interest [1]. Several adipocyte-targeted *Cre* transgenic mouse models have been created to facilitate the study of adipose tissue biology, function, and regulation. The *Fabp4* (also called aP2[2],) promoter-driven *Cre* mouse model was initially widely used to generate adipocyte targeted knockout mouse models but suffered from gene deletion in cells other than adipocytes, sometimes with dramatic effects [3,4]. Further attempts to create a more adipocyte-specific *Cre* transgenic mouse model used the adiponectin (*Adipoq*) gene promoter to drive *Cre* expression. Two groups created *Adipoq-Cre* mouse models, with one using a bacterial artificial chromosome (BAC) transgene [5] and the other using a 5.4-kb promoter fragment to drive *Cre* expression. These models demonstrated greatly improved selectivity for adipocytes and the model using the BAC transgene has gained wide use, with higher levels of observed recombination in adipocytes compared to the 5.4-kb promoter fragment driven *Adipoq-Cre* model [6], and public availability through the Jackson Laboratory mouse repository (Stock #028020).

In the process of developing a new mouse model utilizing the publicly available *Adipoq-Cre* transgenic mouse we discovered strong genetic linkage between our gene of interest, *Adam10*, and the *Adipoq-Cre* transgene. This prompted our investigations to identify the location of the transgenic insertion site and any possible functional consequences of the genetic change. We identified the genomic insertion site of the *Adipoq-Cre* transgene, characterized passenger transgene insertion genes, studied adipose tissue transcriptome changes, and the response of *Adipoq-Cre* transgenic mice to high-fat diet (HFD) feeding.

## 2. Materials and methods

### 2.1. Mice

All animal experiments were performed under the guidelines established by the Institutional Animal Care and Use Committee (IACUC) of Virginia Commonwealth University. Mice were housed in environmentally controlled conditions with a 12-h light/dark cycle (0600 lights on; 1800 lights off) and had free access to food and water. Breeding colonies and experimental mice were maintained in a non-barrier facility with Teklad (Indianapolis, IN) non-irradiated diet 7019. *Adipoq*-Cre mice (C57Bl/6/J background) were purchased from The Jackson Laboratory (Stock #028020) and then maintained and bred in house. These mice were also crossed to *Adam10* floxed mice (C57Bl/6/J background) [7].

### 2.2. Genotyping

Tail DNA was prepared using the following procedure adapted from a previously described method [8]. First, a 2–3 mm section of the tail tip was placed in a 1.5 mL tube, and 200 µL of 25 mM NaOH/0.2 mM EDTA (pH = 12) was added to the tube. Next, the tail in the basic solution was mixed at 1200 rpm at 98°C on a thermomixer for 35 minutes. 200 µL of 40 mM Tris HCl (pH = 5) was then added to the tube. The neutralized tail DNA solution was diluted 50-fold immediately prior to use, and this diluted solution was used as a template for genotyping reactions. PCR reactions consisted of 1X PowerUp SYBR Green Master Mix (Thermo Fisher Scientific, Waltham, MA), 0.1–0.5 μM forward and reverse primers depending on the genotyping assay, 10–20 ng crude tail genomic DNA in a total reaction volume of 10 μL. Reactions were amplified on a QuantStudio 3 real-time PCR system (Thermo Fisher Scientific). The instrument cover was set to 105°C and the PCR program included a hold stage (1 cycle: 50°C/2 min, 95°C/2 min), a PCR stage (35 cycles: 95°C/1 sec, 60°C/45 sec with data collection), and a final melting curve stage (95°C/1 sec, 60°C/20 sec, 95°C/1 sec with a temperature transition of 0.15°C/sec and continuous data acquisition during the melting transition). Data were analysed using the Thermo Fisher Cloud analysis platform, with manual assessment of PCR amplification plots and melting curves by comparison to historical control PCR reactions and expected PCR product melting temperatures. Genotyping primers are shown in **Supplemental Table 1.**

### 2.3. Quantitative PCR, droplet digital PCR, primer design and sequences, and sequencing

Total RNA was extracted using TRIzol (Thermo Fisher Scientific) and cDNA was obtained from 1 μg of RNA using the iScript cDNA Synthesis Kit (Bio-Rad, Hercules, CA). DNA was isolated using the PureLink Genomic DNA Mini Kit (Thermo Fisher Scientific) with optional RNase A digestion. Quantitative PCR was performed using target-specific primers. PCR reactions consisted of 1X PowerUp SYBR Green Master Mix (Thermo Fisher Scientific), 0.5 μM forward and reverse primers, 5–20 ng cDNA (mRNA analysis) or 25–50 ng DNA (mitochondrial or genomic DNA analysis) in a total reaction volume of 10 μL. Reactions were amplified as technical duplicates or triplicates on a QuantStudio 3 real-time PCR system (Thermo Fisher Scientific). The instrument cover was set to 105°C and the PCR program included a hold stage (1 cycle: 50°C/2 min, 95°C/2 min), a PCR stage (45 cycles: 95°C/1 sec, 60°C/20 sec with data collection), and a final melting curve stage (95°C/1 sec, 60°C/20 sec, 95°C/1 sec with a temperature transition of 0.15°C/sec and continuous data acquisition during the melting transition). Data were analysed using the Thermo Fisher Cloud analysis platform with an efficiency-corrected relative quantification (2^ΔΔCq) methodology utilizing *Tbp* as a reference gene for mRNA analysis [9].

Droplet digital PCR (ddPCR) was performed using the QX200 AutoDG Droplet Digital PCR System (Bio-Rad) as per manufacturer’s protocol and published methods [10]. All reaction preparation was performed in a dedicated PCR hood. ddPCR runs included negative template controls and were run in triplicate. Thresholds were manually set for each sample using acceptance criteria defined during the optimization of each assay. The QuantaSoft Analysis software V1.0 was used to assign positive/negative droplets and convert to a copies per mL format. This data was then normalized to *Rpp30* and presented as copies per genome.

Primers were designed using NCBI Primer-BLAST, with selection for ideal target specificity, PCR product size (less than 200 bp, and when possible less than 100 bp), primer annealing temperature (60°C), and PCR product melting temperature (less than 93°C). When possible, primers were designed to either span exon/intron junctions or have an intervening intron greater than 1000 base-pairs. Primers were experimentally validated for mRNA and target specificity and optimized for efficiency (92–99%) across 6-logs input template concentration. Primer panel sequences are listed in Supplemental Table 2. Sequencing analysis is described in Supplemental Methods.

### 2.4. Diet-Induced-Obesity (DIO)

Starting at 8–10 weeks of age, mice were maintained on a chow diet (Teklad 7019) or started on a high-fat diet (HFD) (60% kcal%, D12492, Research Diet Inc., New Brunswick, NJ). Baseline body weights were recorded, and subsequent body weights determined every 1 week or every 4 weeks of diet feeding as indicated in figures. For HFD, 3–4 food pellets were maintained on the cage floor to encourage sustained weight gain. Diet and animal welfare were monitored every 48–72 hours, paying attention to diet levels and food availability. Body weights were tracked for 12–16 weeks of diet feeding.

### 2.5 Intraperitoneal Glucose Tolerance Testing (IPGTT)

Initial glucose tolerance testing was performed on 10 to 12-week-old mice (baseline) and repeated after 12–14 weeks of dietary intervention (continued chow feeding or HFD feeding). Mice were fasted overnight for approximately 16 hours by transferring mice to clean cages with no food but continued access to drinking water. The following day mice were weighed and an approximately 3 mm section of tail removed with sterile surgical scissors immediately prior to glucose tolerance testing. The first drop of tail blood was discarded on laboratory tissue and a second drop of tail blood was expressed and placed on an AimStrip® Plus Blood Glucose Test Strip (Germaine Laboratories, Inc., San Antonio, Texas) in combination with an AimStrip® Plus Blood Glucose Metre (20 to 600 mg/dL glucose range, Germaine Laboratories, Inc.). This data point was the baseline glucose level (t = 0 min). A 10% w/w solution of D-glucose was made by diluting stock D-glucose (45% w/w D-glucose in H_2_O, G8769 Sigma-Aldrich, St. Louis, MO) in normal saline (114–055-721, Quality Biological, Gaithersburg, MD). The 10% glucose solution was warmed for 30 minutes in a 37°C bead bath prior to syringe loading (26 G tuberculin syringe, 1 mL, BD30962, Becton Dickinson, Franklin Lakes, NJ). Mice were injected intraperitoneally (IP) with the volume of 10% w/w glucose injected (μl) = 10 x body weight (g), which is equivalent to 1 g of glucose/kg of body weight. Tail blood glucose levels were measured at 15, 30, 60 and 120 minutes (t = 15, t = 30, t = 60 and t = 120) after glucose injection. Prior to each measurement a laboratory tissue was used to remove the blood clot and fresh blood expressed by gently massaging the mouse tail as necessary. Measurements that exceeded the upper limit of detection were recorded as the limit, 600 mg/dL. At the end of the glucose tolerance testing, mice were returned to clean cages with water and food available *ad libitum* and monitored carefully for health and well-being.

### 2.6. Statistical analysis and data deposition

Statistical comparisons for two-group means were made using the unpaired *t*-test with GraphPad Prism 8.3 (San Diego, CA). Other statistical tests are described in the text or associated figures. Where appropriate, significance was adjusted for multiple comparisons and noted in figures. Statistical significance was accepted at a *p*-value of less than 0.05. *P*-values are represented as *p < 0.05; **p < 0.01; ***p < 0.001; ****p < 0.0001; respectively.

Raw sequencing data has been deposited in an NCBI BioProject with BioProject ID # PRJNA699608.

## 3. Results

### 3.1. Adam10 and the Adipoq-Cre transgene are linked

Initial breeding was able to generate hemizygous *Adipoq-Cre*^±^;*Adam10*^FLOX/WT^ mice during the first breeding cross between the *Adipoq-Cre* transgenic mouse and *Adam10*^FLOX/FLOX^ mice with the resulting pups being born at expected mendelian ratios (data not shown). However, in subsequent crosses to generate an adipocyte-specific conditional deletion model of *Adam10*, there was a failure to generate any hemizygous *Adipoq-Cre*^±^; homozygous *Adam10*^FLOX/FLOX^ mice. With the cause for this observation unknown, we initially hypothesized that deletion of *Adam10* in adipocytes might result in embryonic lethality, or early pup death, similar to models in which *Adam10* is deleted globally [11]. However, after considerable breeding effort, a hemizygous *Adipoq-Cre*^±^; homozygous *Adam10*^FLOX/FLOX^ mouse was born and surprisingly did not have any overt phenotypic changes compared to littermates. In subsequent breeding utilizing this mouse, pups were born with genotypes at the expected frequency, suggesting genetic linkage between the *Adipoq-Cre* transgene and the *Adam10* locus. We initially tested this hypothesis using an informative mating scheme (Supplemental Figure 1) and recorded the resulting genotypes (Table 1). The proportion of hemizygous *Adipoq-Cre*^±^; homozygous *Adam10*^FLOX/FLOX^ mice born was much less than expected (2.2% versus 25%) and statistical testing for linkage [12] was definitive with an estimated genetic distance of 4.4 centimorgans (cM) between the *Adipoq-Cre* transgene and the floxed *Adam10* loci (Table 1). These results demonstrated strong genetic linkage between the *Adipoq-Cre* transgene and *Adam10*.

**Table 1.** Linkage analysis results between the *Adipoq-Cre* transgene and the *Adam10* locus

	Genotypes			
	Cre+; F/F	Cre+; F/W	Cre-; F/F	Cre-; F/W
Expected (%)	25	25	25	25
Actual (%)	2.2	47.3	47.3	2.2
n = 91	2	43	43	2
Theta^a^:	0.044			
Genetic Distance (cM):	4.4			
LOD Score (Z)^b,c^:	25.6			

^a^theta = recombination frequency

^b^LOD Score (Z) = log_10_[[(theta^0)*((1-theta)^n)]/[((1/2)^0)*((1/2)^n)]]

^c^LOD ≥ 3; definitive evidence two loci are linked

### 3.2. Identification of the Adipoq-Cre transgene insertion site

**Figure 1.**
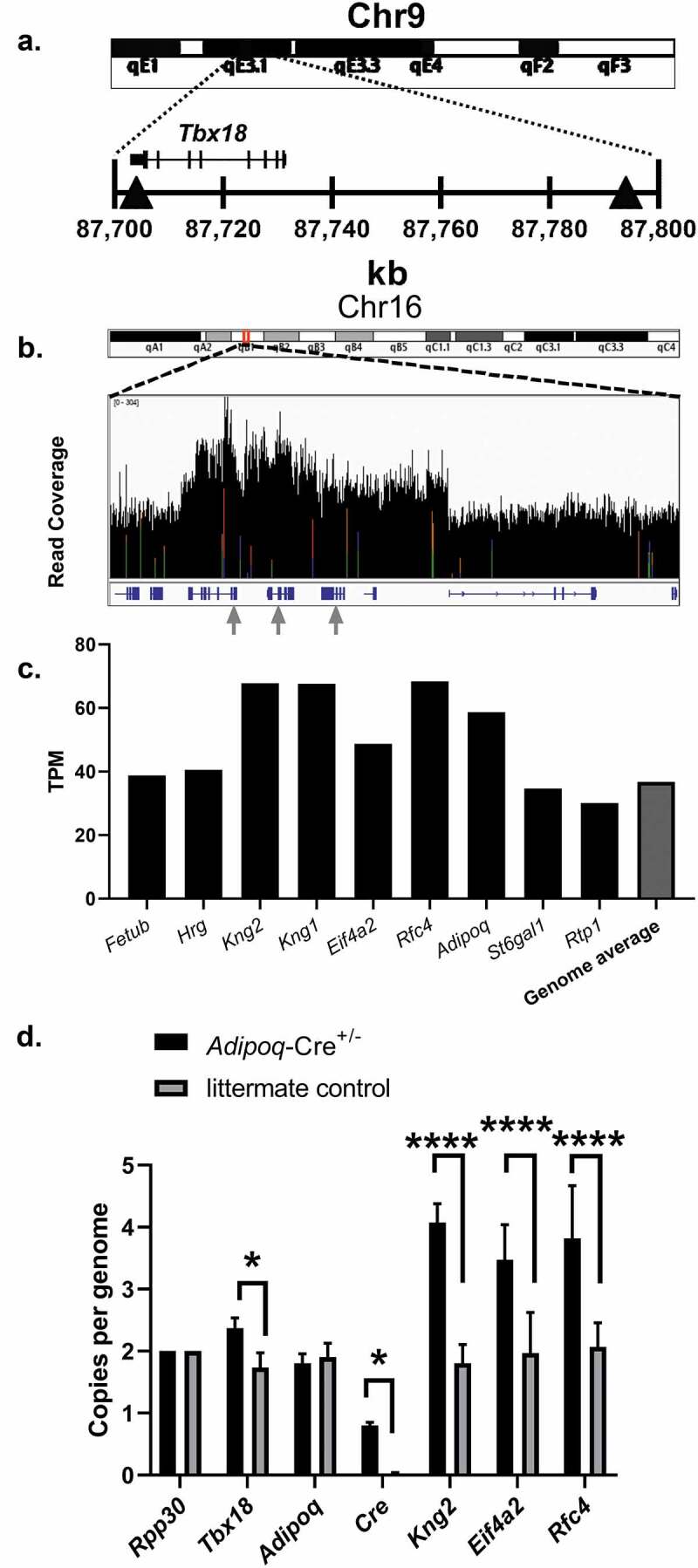
Identification of the *Adipoq-Cre* transgene insertion site and estimation of passenger gene copy number. (a) Abbreviated ideogram of mouse chromosome 9 with red highlighted region in 9qE3.1 expanded to show the genomic context surrounding the gene *Tbx18*. The grey triangles indicate locations where reads mapped to mouse chromosome 16 and had strong evidence for insertion of the *Adipoq-Cre* transgene. (b) Abbreviated ideogram of mouse chromosome 16 with red highlighted region in 16qB1 expanded to show sequencing read coverage (0–304 reads) of genes close to the native *Adipoq* gene locus and were likely included in the BAC used for transgene construction. Genes are shown in blue below the read coverage graph. Gray arrows highlight locations where reads mapped to mouse chromosome 9 near or within the gene *Tbx18* and correspond from left-to-right with genes *Kng2* and *Kng1* (map to far-right triangle in A), and *Rfc4* (maps to exon 8 of *Tbx18*). (c) Quantification of genomic DNA sequencing reads that aligned to the coding sequence of genes which are close to the native *Adipoq* gene. Genome average is the average of 27,180 genes. kb, kilobase. TPM, transcripts per million. Results are from whole genome sequencing of an 8-week-old male *Adipoq-Cre*^±^ mouse. (d) ddPCR results are shown normalized to copies per genome. All values are mean + SD (n = 3/group); ns, not significant; *p < 0.05;****p < 0.0001

Motivated by a desire to locate the *Adipoq-Cre* transgene insertion site, we performed whole-genome sequencing on a hemizygous *Adipoq-Cre*^±^;*Adam10*^WT/WT^ mouse. The data from 100-bp paired end sequencing yielded greater than 80X average coverage of the mouse genome (2,189,169,434 total clean reads) with a 99.28% alignment rate using the sequence aligner Bowtie2 [13]. The mouse genome aligned data (BAM) was analysed using the program LUMPY, a probabilistic framework for structural variant discovery [14]. Screened variants that included the region near the *Adipoq* locus on chromosome 16 and had a split-read or a mate pair that mapped to chromosome 9 were investigated further. Chromosome breakpoints and read specific evidence were visualized and confirmed using the Integrative Genomics Viewer [15–17]. Strong evidence was found for the following structural variants: +[chr16:23,118,416]:-[chr9:87,704,340], +[chr16: 23,025,572]:+[chr9: 87,794,649], +[chr16: 23,070,260]:-[chr9: 87,794,381], where + and – denote chromosome strand orientation. To our surprise, these structural variants between chromosome 9 and chromosome 16 bracketed the transcription factor gene T-box18 (*Tbx18*) on chromosome 9, with one breakpoint located in the final exon of *Tbx18* (Figure 1a). Our results further suggested possible *Tbx18* gene expression changes that might have implications for data generated using the *Adipoq-Cre* transgenic mouse model. Furthermore, the identified *Adipoq-Cre* transgene insertion site is in close genetic proximity to relevant genes in adipocyte biology, including the long-chain fatty-acid elongation genes *Elovl5* and *Elovl4* and the classic brown adipocyte marker gene zinc finger protein of the cerebellum 1 (*Zic1*) (Table 2). It is important to note that it would be difficult or impossible to generate conditional deletion models for these genes using the *Adipoq-Cre* mouse model.

**Table 2.** Selected genes which are near the *Adipoq-Cre* transgene insertion

NCBI Gene ID	Gene Name	Genetic Distance (cM)	Chr9 Location (NC_000075.6)
11,487	*Adam10*	39.53	(70,678,944.70780229)
68,801	*Elovl5*	43.36	(77,917,365.77984519)
83,603	*Elovl4*	45.6	(83,778,692.83806305, complement)
76,365	*Tbx18*	47.06	(87,702,800.87731260, complement)
22,771	*Zic1*	48.26	(91,358,058.91366247, complement)

Chromosome 9 genes which are in close genetic proximity to the *Adipoq-Cre* transgene insertion site within the gene *Tbx18*, are important in adipocyte biology, and will be difficult or impossible to generate conditional deletion models using the *Adipoq-Cre* mouse model.

### 3.3. Passenger gene insertion and copy number estimation

The *Adipoq-Cre* transgenic mouse model [5] was generated using a 245 kb mouse BAC (RP23-90G21) containing the *Adipoq* gene and several other mouse genes. The start site and 222 bp of the *Adipoq* gene were replaced with the *Cre* recombinase coding sequence in the BAC. It is not known whether copies of the *Adipoq-Cre* transgene were inserted into multiple chromosomes during generation of the mouse line, but extensive backcrossing onto the C57BL/6 J strain has now fixed the transgenic insertion site to chromosome 9. To investigate the *Adipoq-Cre* transgene further, we next identified the additional insertion of passenger genes which were alongside the *Adipoq-Cre* transgene in the BAC construct. Aligned sequencing data were explored using the Integrative Genomics Viewer and a region surrounding the *Adipoq* gene locus was viewed to determine read coverage and identify potential gene copy number increases due to the *Adipoq-Cre* transgene (Figure 1b). The identified genes with likely copy number changes were, kininogen 2 (*Kng2*), kininogen 1 (*Kng1*), eukaryotic translation initiation factor 4A2 (*Eif4a2*), replication factor C (activator 1) 4 (*Rfc4*), and *Adipoq*. We next used featureCounts [18], a read summarization program to count sequencing reads to each identified gene in order to quantify read coverage for each gene. The results showed increased gene-wide reads for the genes *Kng2, Kng1, Eif4a2, Rfc4*, and *Adipoq* compared to neighbouring genes and the average of the entire mouse genome (Figure 1c). Primers were designed and optimized to confirm passenger gene copy number and gene expression using droplet digital PCR [10] and are shown in Supplemental Table 2. Copy number analysis using ddPCR showed increased copy number for the genes *Kng2, Eif4a2*, and *Rfc4* (Figure 1d). Primers designed to determine copy number for *Tbx18* additionally showed a significant increase (Figure 1d).

### 3.4. Adipose tissue transcriptome analysis

**Figure 2.**
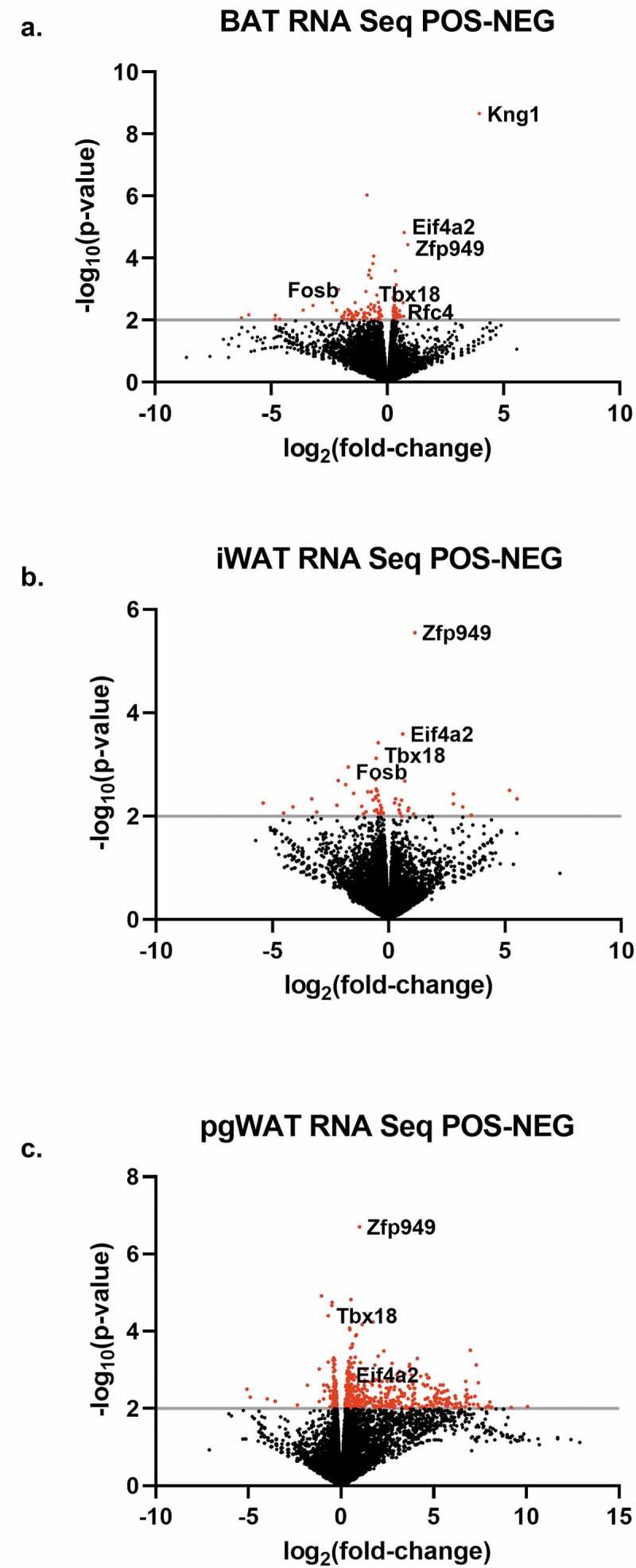
**Adipose tissue differential gene expression analysis**. Transcriptional changes were assessed between 8-week-old male *Adipoq-Cre*^±^ mice and *Cre*-negative littermate controls using mRNA sequencing. Differential expressed genes with a *p*-value less than 0.01 (grey line) are identified in red, above all other genes which are black. (a) Brown adipose tissue, (b) inguinal white adipose tissue, (c) perigonadal white adipose tissue. Representative genes are shown. n = 4/group

We next performed RNA sequencing analysis on interscapular brown adipose tissue (BAT), inguinal white adipose tissue (iWAT) and perigonadal white adipose tissue (pgWAT) isolated from 8-week-old male hemizygous *Adipoq-Cre*^±^ mice and *Cre*-negative littermate controls to investigate transcriptome changes introduced by the *Adipo-Cre* transgene. Transcriptional changes were overall low with 115 genes for BAT (Figure 2a), 53 genes for iWAT (Figure 2b) and 515 genes for pgWAT (Figure 2c) meeting criteria for altered gene expression. Furthermore, gene ontology and pathway analysis failed to find significant gene enrichment (data not shown). However, differential gene expression analysis did reveal gene expression changes in the previously identified transgene passenger genes *Kng1, Eif4a2* and *Rfc4*; as well as *Tbx18* and the gene zinc finger protein 949 (*Zfp949*). *Zfp949* expression was elevated in all three tissues and it is worth noting that this gene is downstream of the *Adipoq-Cre* transgene insertion, suggesting possible long-range alteration of *Zfp949* regulation.

**Figure 3.**
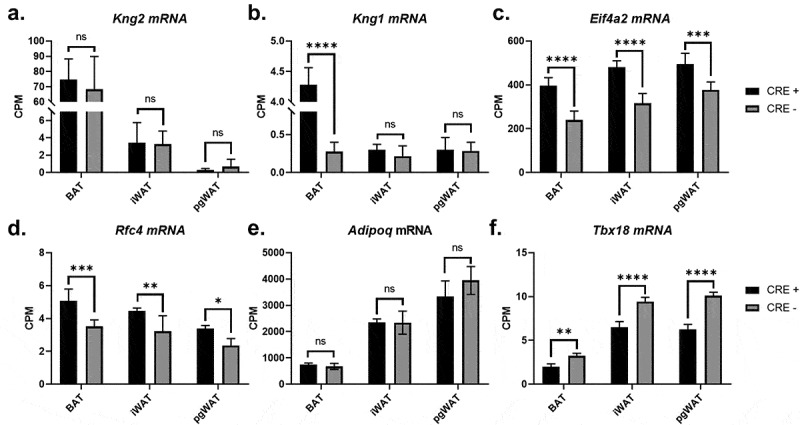
*Adipoq-Cre* transgenic mouse passenger gene expression analysis. Transcriptional changes were assessed between 8-week-old male *Adipoq-Cre*^±^ mice (black bars) and *Cre*-negative littermate controls (grey bars) in the genes (a) *Kng2*, (b) *Kng1*, (c) *Eif4a2*, (d) *Rfc4*, (e) *Adipoq* and (f) *Tbx18*, revealing increased gene expression in several passenger genes and a consistent decrease in *Tbx18* gene expression due to transgene insertion in the native *Tbx18* locus. BAT, brown adipose tissue; iWAT, inguinal white adipose tissue; pgWAT, perigonadal white adipose tissue. CPM, normalized counts per million. All values are mean ± SD (n = 4/group); ns, not significant; *p < 0.05; **p < 0.01; ***p < 0.001; ****p < 0.0001

The transgene passenger gene *Kng2* did not have altered gene expression (Figure 3a) and the neighbouring gene *Kng1* only had gene expression changes in BAT, with an approximately 15-fold higher expression in BAT, though total expression was low (Figure 3b). The remaining passenger genes *Eif4a2* and *Rfc4* had gene expression that was consistently and significantly elevated in BAT, iWAT and pgWAT (Figure 3(c-d)), and these changes may potentiate the differential gene expression changes identified in the adipose tissues. *Adipoq* gene expression was not altered in any of the adipose tissues (Figure 3e) which is consistent with insertion of the *Cre* recombinase coding sequence into the start codon of *Adipoq* within the *Adipoq-Cre* transgene. *Tbx18* gene expression was reduced 30–40%, providing additional evidence for the disruption of one copy of the *Tbx18* gene on chromosome 9 by the *Adipoq-Cre* transgene (figure 3f). As the expression of *Tbx18* is important in development [19], gene expression changes were determined for *Tbx18* and BAC passenger genes in heart, kidney, liver, and brain. Additionally, heart and liver weight were assessed. No differences were found in organ weight or gene expression except *Kng2* was significantly increased in the kidney (Supplemental Figure 2A-E).

### 3.5. The Adipoq-Cre transgene does not alter high-fat diet-induced obesity and glucose tolerance

**Figure 4.**
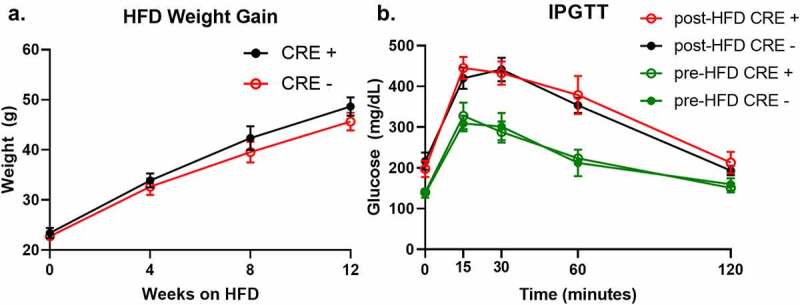
Mice hemizygous for the *Adipoq-Cre* transgene have similar weight gain and glucose tolerance after HFD feeding compared to littermate controls lacking the *Adipoq-Cre* transgene. (a) Body weights of *Adipoq-Cre*^±^ (red, open circles) and control littermates (black, solid circles) starting at 12 weeks old and throughout 12 weeks of HFD feeding (n = 5/group). (b) Intraperitoneal glucose tolerance tests (IPGTT) performed on *Adipoq-Cre*^±^ (green and red open circles) and control littermates (green and black solid circles) starting at 12 weeks old (baseline, green) and after 12 weeks of HFD feeding (n = 5/group). All genotype comparisons were not significant. Values are means ± SEM

As a final investigation, we assessed whether the changes introduced by the *Adipo-Cre* transgene could influence high-fat diet (HFD) induced obesity and carbohydrate metabolism tolerance. Weight gain during 12 weeks of HFD feeding was similar between *Adipoq-Cre*^±^ mice and *Cre*-negative littermate controls (Figure 4a). Prior to starting HFD, initial glucose tolerance was tested in 12-week-old *Adipoq-Cre*^±^ and *Cre*-negative males and no changes were observed between the groups (Figure 4b). Furthermore, no changes were observed in glucose tolerance between *Adipoq-Cre*^±^ mice and *Cre*-negative controls after 12-weeks of HFD feeding, suggesting that any changes introduced by the *Adipoq-Cre* transgene have a limited effect on HFD-induced weight gain and glucose tolerance.

## 4. Discussion

A recent report [20] highlights the need for further characterization of transgenic *Cre* mouse lines, observing that only 36/1631 (2.3%) *Cre* models published in the Mouse Genome Database (MGD[21],) have an annotated chromosome insertion location. Furthermore, the authors of this report discovered that insertion sites disrupt the coding sequence of endogenous genes in 21/40 (52.5%) analysed transgenic mouse lines, with frequent large deletions and/or structural variations at the insertion site [20]. These results demonstrate the importance of identifying *Cre* transgene integration sites and the impact this information can have on study results obtained using the mouse models. A variety of approaches can be used to identify transgene insertion sites and include low-resolution cytogenetic approaches such as fluorescence in-situ hybridization (FISH) [22] and higher resolution molecular approaches such as targeted locus amplification (TLA) [23] and highly parallel DNA sequencing with or without paired reads [24,25].

In this report, we serendipitously identified the *Adipoq-Cre* transgenic chromosome insertion site during initial generation of an adipocyte-specific deletion model of the gene *Adam10*. This preliminary discovery by ‘old-fashioned’ linkage analysis prompted our further investigations into the genomic and transcriptional changes introduced by the *Adipoq-Cre* transgene. Whole-genome sequencing of the *Adipoq-Cre* transgenic mouse model revealed both the location of the transgene insertion and passenger genes that were introduced with the *Adipoq-Cre* BAC. Innovation and cost-saving strategies in highly parallel sequencing continue to reduce the cost of whole-genome sequencing data generation and make this approach increasingly appropriate when characterizing new transgenic mouse models.

Our results indicate that the *Adipoq-Cre* transgene inserted within the *Tbx18* gene locus on chromosome 9 and this insertion causes a significant decrease in *Tbx18* gene expression in adipose tissue. *Tbx18* expressing myocardial progenitor cells give rise to the epicardium, cardiac fibroblasts, and coronary smooth muscle cells [26] and mouse embryos without *Tbx18* have structural and functional defects in the epicardium and coronary vessels [27]. *Tbx18* expression is critical for normal mouse development and mice lacking *Tbx18* die shortly after birth as a result of severe skeletal malformations [19]. These data suggest that mice homozygous for the *Adipoq-Cre* transgene may not be viable or may suffer from abnormalities resulting from the alteration of the native *Tbx18* gene.

We identified additional insertion of genes *Kng2, Kng1, Eif4a2* and *Rfc4* that were contained in the BAC used for *Adipoq-Cre* transgene construction. These passenger genes may have functional consequences in various tissues throughout the mouse, as their expression is not limited to adipocytes. The impacts of increased passenger gene expression are difficult to predict, but tissue-specific expression changes are expected, and these may have implications for any study of adipose tissue physiology. Tissue-specific gene expression data from the ENCODE project [28] for each passenger gene, and *Tbx18* and *Adipoq* are shown in Supplemental Figure 3 A-F.

Adipose tissue transcriptome analysis in this study was limited to young chow-fed male mice and reflects differential gene expression changes in adipose tissue, and not adipocytes alone. Primary culture and differentiation of adipocyte precursors, or single nuclei adipocyte RNA sequencing [29] could be used to identify adipocyte-specific transcriptional changes due to the presence of passenger genes and *Tbx18* alteration. Transcriptional differences between *Adipoq-Cre*^±^ mice and *Cre*-negative littermate controls may be more pronounced in the HFD state. We did not analyse adipose tissue following HFD and recommend careful interpretation of data from gene deletion models using the *Adipoq-Cre* transgenic mouse in this setting.

Despite the genomic and gene expression changes observed in the *Adipoq-Cre* transgenic mouse, mice hemizygous for the *Adipoq-Cre* transgene did not have a significant difference in weight gain trajectory or glucose homoeostasis after 12 weeks of HFD feeding. These results suggest that the presence of the *Adipoq-Cre* transgene has no or only a limited effect when the outcome is weight gain or carbohydrate metabolism. Nevertheless, our results do not exclude the possibility of gene interaction effects that may have metabolic consequences between the *Adipoq-Cre* transgene and the conditional deletion gene of interest. It is important to note that such interaction effects are difficult to test in any breeding strategy using *Adipoq-Cre* transgenic mice and a conditional deletion gene of interest.

We attempted local *de-novo* assembly of the transgenic insertion but were unsuccessful (data not shown). Our inability to generate an assembly for the transgenic insertion likely results from two limitations in our approach. The first is our use of short read sequencing alone (100-bp paired end), and the second is our choice to sequence the genomic DNA of a hemizygous *Adipoq-Cre*^±^ mouse. The first limitation can be overcome by targeted sequencing after identification of the transgenic insertion region with traditional chain terminator sequencing alone or in combination with long-read sequencing [30]. Both approaches add considerable effort and cost but may be necessary depending on the scientific question and model generated. With regards to the second limitation, genomic DNA sequencing of mice homozygous for a *Cre* transgene may not be possible due to the *Cre* transgene being targeted to the native promoter of a cell- or tissue-specific gene that is required for mouse development or survival. With regards to the *Adipoq-Cre* transgenic mouse, we chose not to attempt breeding the mouse strain to a homozygous state as this was not attempted by the donating investigator and would have presented difficulties in properly genotyping the resulting mice (one copy versus two copies), and may have resulted in lethality due to *Tbx18* gene alteration. An attempt to generate a homozygous animal may provide additional insight into the model. We nevertheless demonstrated that identification of the *Cre* transgene insertion can be made in hemizygous *Cre* transgenic mice, with the potential to generalize our approach to other *Cre* transgenic mice.

## 5. Conclusions

We have identified the genomic insertion site of the *Adipoq-Cre* mouse transgene, and this finding has important implications for the use of this mouse model in studies of adipocyte biology. The identification of the *Adipoq-Cre* transgene insertion site was facilitated by initial localization through linkage analysis with the *Adam10* locus. The approach used in our study is however generalizable to the identification of other transgene insertion sites but may be less successful when there are multiple transgene insertion sites throughout the genome. In the ideal situation, the *Cre* transgene is limited to a single insertion site and this prevents unwanted phenotypic drift in model systems that use the *Cre* transgenic mouse. *Cre*-driver lines are best when the transgene location is known, the transgene is inherited as a single unit, *Cre*-expression is appropriate, and passenger genes in the transgenic construct are limited. Highly parallel DNA sequencing provides an excellent tool for identifying or confirming transgene insertion and choosing founders which have these characteristics. We hope that this approach will be used more often in the future to help prevent unwanted consequences of transgene insertion, to better understand the advantages and limitations of the model developed and promote reproducible scientific inquiry.

## Supplementary Material

Supplemental Material

## Highlights

- The genomic insertion site of the adiponectin-cre transgene is on chromosome 9.
- The genomic insertion site of the adiponectin-cre transgene disrupts the *Tbx18* gene locus.
- Passenger gene insertion occurred with the generation of the *Adipoq-Cre* transgenic mouse and these passenger genes could have functional consequences.
