# Supplementary material for: Identification of the transgene insertion site for an adipocyte-specific adiponectin-cre model and characterization of the functional consequences
Source: Adipocyte. 2021 Feb 10;10(1):91–100. doi: 10.1080/21623945.2021.1880083 (PMC7889145; doi:10.1080/21623945.2021.1880083)
Supplement: Supplemental Material [file KADI_A_1880083_SM8703.zip › Supplementary information/Supplemental Figures Adipoq Insertion Paper.docx]

Supplemental Table 1 Genotyping Primers

| Primer Name | Primer Sequence | Target | Purpose | Final Primer Concentration (μM) |
| --- | --- | --- | --- | --- |
| F_Cre Melt | AAATGCTTCTGTCCGTTTGC | Cre | Cre Genotyping | 0.5 |
| R_Cre Melt | ATGTTTAGCTGGCCCAAATG | Cre | Cre Genotyping | 0.5 |
| F_Cre IPC | ATCTGAAAGACAAGAAACAGGGGA | Internal Positive Control | Cre Genotyping | 0.1 |
| R_Cre IPC | CACTACAGACCCATGAGGAGTTC | Internal Positive Control | Cre Genotyping | 0.1 |
| F_Adam10 | TGCCAGTACATAGTGATGCAATTGATTAT | Adam10 | Adam10 Genotyping | 0.5 |
| R_Adam10 | CAAAACTACCCTCCCACAAAGGA | Adam10 | Adam10 Genotyping | 0.5 |

| Supplemental Table 2. Primers for validation of Adipoq cre transgene passenger gene expression and copy number   \| **Gene** \| **NCBI Gene ID** \| **Full Gene Name** \| **Forward Primer (5'-3')** \| **Reverse Primer (5'-3')** \| **Product Length (bp)** \| **PCR Efficiency** \| \| --- \| --- \| --- \| --- \| --- \| --- \| --- \| \| *Adipoq* \| 11450 \| adiponectin, C1Q and collagen domain containing(Adipoq) \| TGACGACACCAAAAGGGCTC \| CACAAGTTCCCTTGGGTGGA \| 141 \| 102 \| \| *Adipoq_gDNA* \| 11450 \| adiponectin, C1Q and collagen domain containing(Adipoq) \| CCCAGTCATGCCGAAGATGA \| CACAAGTTCCCTTGGGTGGA \| 79 \| 99 \| \| *Eif4a2* \| 13682 \| eukaryotic translation initiation factor 4A2(Eif4a2) \| TAGTATTGGCCCCCACCAGA \| CCTTTTGGATCTGTTGAGCCAG \| 45 \| 102 \| \| *Eif4a2_gDNA* \| 13682 \| eukaryotic translation initiation factor 4A2(Eif4a2) \| CCCCTCACATTGTTGTTGGTACT \| TTTAGCATATCAAACACTCTCCCTGG \| 49 \| 101 \| \| *Fetub* \| 59083 \| fetuin beta(Fetub) \| TTACAGTGGGAAGACATGGGATCT \| TGCTTTGCACTGGCCATAAAC \| 141 \| 94 \| \| *Fetub_gDNA* \| 59083 \| fetuin beta(Fetub) \| ACCCTGAGACTCCCTAGCAG \| CTGTGTCCTTCCCAATCCCC \| 83 \| 101 \| \| *Hrg* \| 94175 \| histidine-rich glycoprotein(Hrg) \| ACACAGCATTTCCCCAGGTC \| GGGGTTTCCAAGTCAGAGGTT \| 89 \| 99 \| \| *Hrg* \| 94175 \| histidine-rich glycoprotein(Hrg) \| CTGAGTCCCACCAACTGTGA \| TAGTAGACTGTGGCCGTTCC \| 149 \| 100 \| \| *Hrg_gDNA* \| 94175 \| histidine-rich glycoprotein(Hrg) \| AGGTCAGTATCATCGGGGCT \| TCAGTGGAGGGAGTCGGTAG \| 122 \| 98 \| \| *Kng1* \| 16644 \| kininogen 1(Kng1) \| TGACTTCAAGGACGCTGAGG \| GGGCCTTACTTGGAGCAATCT \| 116 \| 102 \| \| *Kng1* \| 16644 \| kininogen 1(Kng1) \| TGCCAAGCATTAGATATGACTGAAA \| CCTGGCAATGTAGGGTGGAC \| 120 \| 98 \| \| *Kng1_gDNA* \| 16644 \| kininogen 1(Kng1) \| GGCTGTATTTCAGGCTGTGGATTT \| AGGGTTAAACTGCTTCAGAGAGA \| 46 \| 101 \| \| *Kng2* \| 385643 \| kininogen 2(Kng2) \| TGACCAAGCGCGCTAAAATG \| CATTCTCCAGTGGCGGCTTC \| 145 \| 97 \| \| *Kng2_gDNA* \| 385643 \| kininogen 2(Kng2) \| GCTAGGTTACAAAGCGGCAAC \| TGGTCACTCGGTACAACACAA \| 46 \| 101 \| \| *Rfc4* \| 106344 \| replication factor C (activator 1) 4(Rfc4) \| CAAAGCACAACTGACCAAGGAC \| GGAAAGCCACTTCATCCACAC \| 128 \| 101 \| \| *Rfc4_gDNA* \| 106344 \| replication factor C (activator 1) 4(Rfc4) \| GCTTGGCAGATGGTGCAGAT \| CATCACAGTTGCACAAAGGCT \| 59 \| 101 \| \| *Rpp30* \| 54364 \| ribonuclease P/MRP 30 subunit(Rpp30) \| ATGACGTGGCAAACTTAGGACT \| CTGCAATTTGTGGACACGGC \| 76 \| 100 \| \| *Rpp30_gDNA* \| 54364 \| ribonuclease P/MRP 30 subunit(Rpp30) \| AATGTGAGGGCTGAGACGAG \| GACTGATGAGCTACGAAGGGG \| 67 \| 101 \| \| *Rtp1_gDNA* \| 239766 \| receptor transporter protein 1 (Rtp1) \| TTGACAAAGGGGTGGCAGTT \| TGCCACTTCACAAGCACTGA \| 70 \| 96 \| \| *St6gal1_gDNA* \| 20440 \| beta galactoside alpha 2,6 sialyltransferase 1 (St6gal1) \| TGTGGGGGTTAGGTCTAGGG \| TCCTGGGCACTCTATCCACA \| 70 \| 101 \| \| *Tbx18* \| 76365 \| T-box18(Tbx18) \| TCCATTTGCCAAAGGTTTCCG \| ATGACTCCACCAGAGCTTCC \| 68 \| 102 \| \| *Tbx18_gDNA* \| 76365 \| T-box18(Tbx18) \| AGCAACCCGTCTGTGAACAT \| GACGGCGATGGCATGATGTA \| 128 \| 101 \| |
| --- | --- | --- | --- | --- | --- | --- | --- | --- | --- | --- | --- | --- | --- | --- | --- | --- | --- | --- | --- | --- | --- | --- | --- | --- | --- | --- | --- | --- | --- | --- | --- | --- | --- | --- | --- | --- | --- | --- | --- | --- | --- | --- | --- | --- | --- | --- | --- | --- | --- | --- | --- | --- | --- | --- | --- | --- | --- | --- | --- | --- | --- | --- | --- | --- | --- | --- | --- | --- | --- | --- | --- | --- | --- | --- | --- | --- | --- | --- | --- | --- | --- | --- | --- | --- | --- | --- | --- | --- | --- | --- | --- | --- | --- | --- | --- | --- | --- | --- | --- | --- | --- | --- | --- | --- | --- | --- | --- | --- | --- | --- | --- | --- | --- | --- | --- | --- | --- | --- | --- | --- | --- | --- | --- | --- | --- | --- | --- | --- | --- | --- | --- | --- | --- | --- | --- | --- | --- | --- | --- | --- | --- | --- | --- | --- | --- | --- | --- | --- | --- | --- | --- | --- | --- | --- | --- | --- | --- | --- | --- | --- | --- |


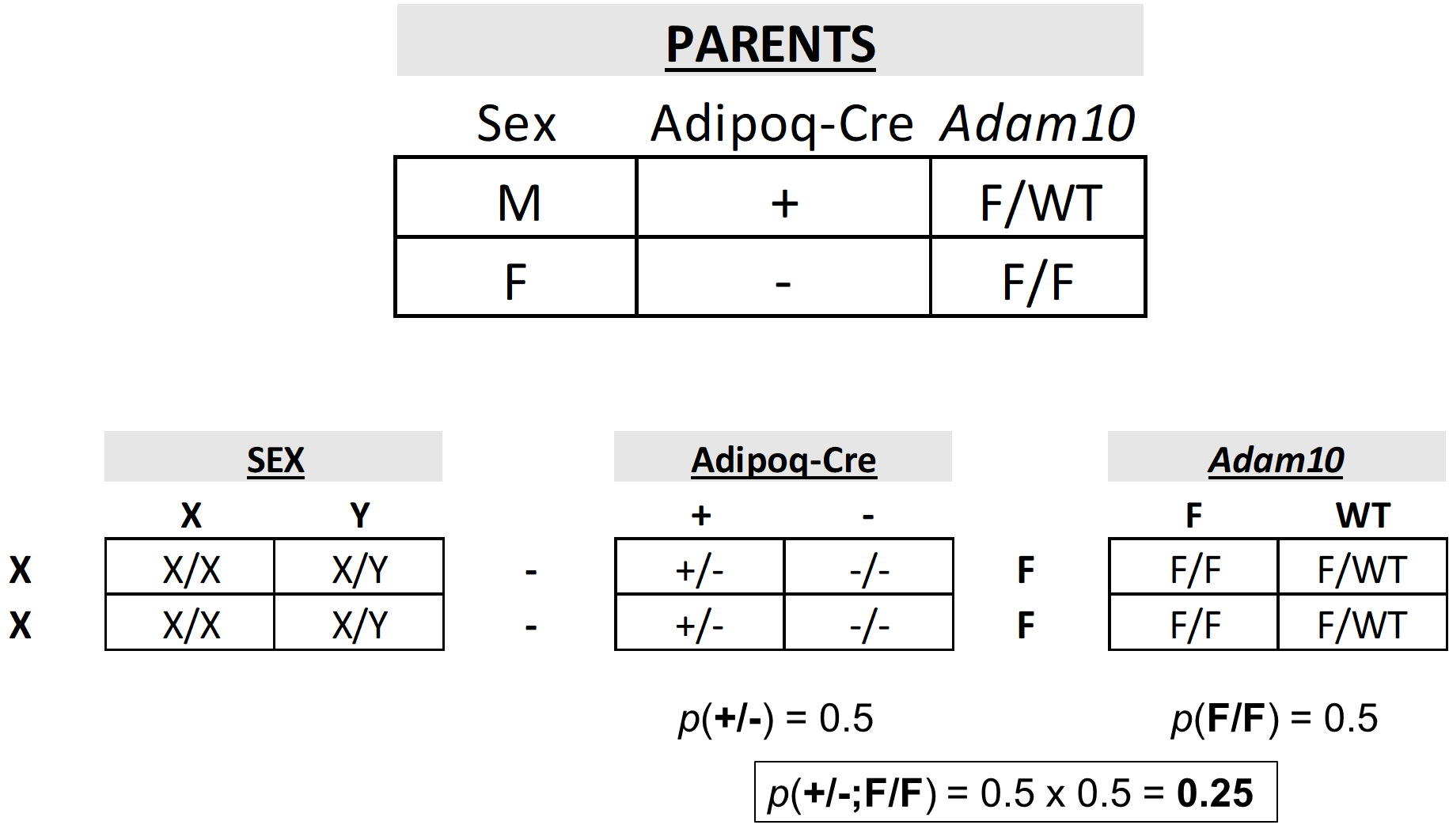


Supplemental Figure 1. Informative mating scheme to determine whether the *Adipoq*-*Cre* transgene and the *Adam10* locus are linked.

One founder (in this instance, the male) is hemizygous for the *Adipoq*-*Cre* transgene and heterozygous for the floxed allele at the *Adam10* locus. The other founder (female) does not carry the *Adipoq*-*Cre* transgene and is homozygous for the floxed allele at the *Adam10* locus. Below the parental genotypes, Punnett squares display predicted allele combinations and the combined probability of generating a progeny that is hemizygous for the *Adipoq*-*Cre* transgene and homozygous for the floxed allele at the *Adam10* locus. In this breeding arrangement it is expected that 25% of progeny, on average, would have this combined genotype.

**A.**

**C.**

**E.**

**B.**

**D.**

**F.**

**Supplemental Figure 2. Gene expression in mouse tissue types.**

(A) *Adipoq*, (B) *Eif4a2*, (C) *Kng1*, (D) *Kng2*, (E) *Rfc4*, (F) *Tbx18* gene expression in mouse tissue types. Mouse ENCODE transcriptome data, profiling developmental and mature tissues, demonstrating high and selective gene expression in the adult genital fat pad, subcutaneous fat pad, and mammary gland. RPKM, reads per kilobase per million reads placed.
