# Supplementary material for: Identification of the transgene insertion site for an adipocyte-specific adiponectin-cre model and characterization of the functional consequences
Source: Adipocyte. 2021 Feb 10;10(1):91–100. doi: 10.1080/21623945.2021.1880083 (PMC7889145; doi:10.1080/21623945.2021.1880083)
Supplement: Supplemental Material [file KADI_A_1880083_SM8703.zip › Supplementary information/Supplemental Methods_Final.docx]

***1. Sequencing Data Analysis Platform***

Data analysis was performed using a cloud-based version of Galaxy (release 18.05, (9)), a virtual bioinformatics analysis environment (https://galaxyproject.org/). In order to establish a virtual instance of Galaxy, the cloud manager CloudMan(10) was used to provision and manage compute clusters in the cloud infrastructure. Amazon Web Services was used for cloud computation and an existing Amazon Machine Image (AMI) of CloudMan was used to launch the instance (ami-3be8cd2c). An Amazon Elastic Compute Cloud (EC2) memory optimized r5.2xlarge instance with an Intel Xeon Platinum 8000 series (Skylake-SP) processor (8 vCPUs), sustained all core Turbo CPU clock speed of up to 3.1 GHz, and 64 GB of computational memory was used for the master instance. The master instance was scalable through CloudMan to include up to 20 worker instances of any computational and memory specification, depending on the requirements of the data analysis being performed. All jobs were set to be performed on worker nodes and not the master instance. The transient storage associated with the master instance was set to 6 TB and was scaled up as needed for data storage. Genomics Virtual Laboratory (GVL, version 4.4.0, (11)), a versatile genomics workbench for Galaxy, RStudio and Jupyter, was used to launch CloudMan and the associated Galaxy workspace. Sequencing data was first uploaded to a secure FTP server (ProFTPD version 1.3.6b) using a local client (FileZilla, version 3.46.3) and then downloaded to the transient storage associated with the master cloud instance. Analysis pipelines were created within Galaxy to analyze sequencing data. All pipelines included an initial quality control check step of the data using FASTQC (Andrews, S. FastQC, A Quality Control tool for High Throughput Sequence Data, The Bioinformatics Group at the Babraham Institute) to confirm adaptor and low-quality read removal prior to initiating data analysis.

***1.2. Whole Mouse Genome Re-sequencing, Library Construction, Sequencing, and Data Analysis.***

Whole genome resequencing of a male hemizygous *Adipoq*-*Cre*^+/-^ mouse was performed by BGI Americas using the DNBseq high-throughput sequencing methodology that utilizes combinatorial Probe-Anchor Synthesis (cPAS), linear isothermal rolling circle amplification (RCA) and DNA Nanoball (DNB^TM^) technology, followed by high-resolution digital imaging(12-14). Linear amplification and the DNB method reduces error rates while simultaneously enhancing signal. The size of the DNB is controlled so that only one DNB is bound per active site of the DNBseq flow cell and the densely patterned array provides increased flow cell utilization and sequencing accuracy.

Genomic DNA was extracted from 100 mg of kidney from an 8-week-old male hemizygous *Adipoq*-*Cre*^+/-^ mouse. DNA concentration was determined by fluorometry (Qubit, Invitrogen) and sample integrity and purity assessed using agarose gel electrophoresis (1% agarose, voltage:150 V, electrophoresis time: 40 min). 1 μg genomic DNA was randomly fragmented using a Covaris.focused-ultrasonicator and the resulting fragmented genomic DNA (average size 200-400 bp) was bound to magnetic beads (Agencourt AMPure XP), DNA fragments were end repaired, 3’ adenylated, and sequencing adaptors ligated to the ends of these 3’ adenylated fragments. Adaptor containing DNA fragments were then amplified by PCR and the products purified using magnetic beads. The double stranded PCR products were heat denatured and circularized using a splint oligonucleotide that was homologous to the adaptor regions of the library to generate a single stranded DNA circle that was closed with a ligation reaction. This was followed by an exonuclease digestion to remove any single-stranded non-circularized DNA. The single stranded circular DNA was the final format of the sequencing library and underwent final linear amplification with phi29 polymerase (Rolling Circle Amplification) to generate 300-500 copies of the library which folded into DNA nanoballs (DNB). The resulting DNB library was quantified by fluorometry.

The DNB library was flowed across a patterned nanoarray with positive surface charged “active sites” that were sized to bind a single DNB. Paired-end 100 bp sequencing was performed using combinatorial Probe-Anchor Synthesis (cPAS), which involves initial hybridization of sequencing primers to the adaptor region of the DNB followed by sequential incorporation of a fluorescently labeled dNTP, imaging of the flow cell, and removal of the fluorescent dye which enables continued sequencing by synthesis cycles. Data output consisted of 2,189,169,434 clean reads (adaptor and low-quality read trimmed), providing an average sequencing coverage of >80X of the mouse genome with an overall 99.28% alignment rate.

Using the cloud-based virtual instance of the Galaxy bioinformatics analysis environment described in the *Sequencing Data Analysis Platform* section, adaptor trimmed, clean reads (FASTQ) were mapped using the fast gapped-read alignment program Bowtie 2 (15) using settings for paired-end reads and the mouse mm10 reference genome. The alignment data output (BAM) from Bowtie2 was loaded in the Integrative Genomics Viewer (IGV 2.6.2,(16-18)) for sequencing data visualization and exploration. A region surrounding the *Adipoq* gene locus was visualized corresponding to Chr16:22,816,658-23,455,665 of the mm10 mouse genome. The coverage track was adjusted to a scale of 0-304 and read coverage visualized within this window for the region selected to determine possible gene amplification due to the *Adipoq*-*Cre* transgene. The alignment data output (BAM) from Bowtie2 was also loaded into the program LUMPY, a probabilistic framework for structural variant discovery (19). The output from LUMPY included all structural variants detected and the read evidence for the calls. The data output from LUMPY was manually sorted by chromosome location and those variants detected in chromosome 16 or chromosome 9 of the mouse genome were selected for further inspection. Screened variants that included the region near the *Adipoq* locus on chromosome 16 and had a split-read or a mate pair that mapped to chromosome 9 were investigated further. Strong evidence was present for the following three structural variants that met these criteria: +[chr16:23118416]:-[chr9:87704340], +[chr16: 23025572]:+[chr9: 87794649], +[chr16: 23070260]:-[chr9: 87794381], where + and – denote chromosome strand orientation. Chromosome breakpoints and read specific evidence was visualized and confirmed in the Integrative Genomics Viewer. The alignment data output (BAM) from Bowtie2 was also loaded into featureCounts (20), a read summarization program that counts sequence reads to each gene in a supplied annotation file. This was done to quantify coding region gene amplification due to the insertion of the *Adipoq*-*Cre* transgene. Exons were defined as features, and genes were defined as meta-features, with counts being summarized at the meta-feature or gene level. Reads or fragments that overlapped multiple genes were excluded from the counts. The featureCounts built-in annotation for the mouse genome, mm10, was used, and an output format producing a tabular file containing counted reads, per gene, per row, with a last column of effective gene-length. From this output data, transcripts per million (TPM) were manually calculated for the genes *Fetub*, *Hrg*, *Kng2*, *Kng1*, *Eif4a2*, *Rfc4*, *Adipoq*, *St6gal1*, *Rtp1*, and the average for all 27,180 genes in the featureCounts output.

***RNA Sequencing, Library Construction, Sequencing, and Data Analysis.***

RNA sequencing of adipose tissue samples was performed by BGI Americas using the DNBseq high-throughput sequencing methodology that utilizes combinatorial Probe-Anchor Synthesis (cPAS), linear isothermal rolling circle amplification (RCA) and DNA Nanoball (DNB^TM^) technology, followed by high-resolution digital imaging(12-14). RNA sequencing provides accurate digital expression profiling by measuring transcript sequence and performing comparative analysis to study the expression pattern of genes. High throughput sequencing can quickly and comprehensively obtain transcript sequence information for a specific sample, making it an excellent tool for gene function discovery in transgenic mouse models.

Adipose tissue was isolated, immediately homogenized in TRIzol (Thermo Fisher Scientific), with total RNA isolated and shipped to BGI in a precipitated form in 70% ethanol for further processing. Total RNA was further processed if it met the following criteria: amount ≥200ng; concentration ≥20ng/μl; RNA integrity number (RIN) ≥7.0; ribosomal RNA (rRNA) 28S/18S ≥1.0; and optical density (OD)260/280 ≥1.8 and OD260/230 ≥1.8. Messenger RNA (mRNA) was purified from total RNA using oligo(dT) capture magnetic beads. Purified mRNA was fragmented enzymatically and first strand complementary DNA (cDNA) generated by random hexamer priming and extension with reverse-transcriptase, followed by second strand cDNA synthesis. cDNA fragments were end repaired, 3’ adenylated, and sequencing adaptors ligated to the ends of these 3’ adenylated fragments. Adaptor containing cDNA fragments were then amplified by PCR and the products purified using magnetic beads (Agencourt AMPure XP). The purified products were validated for proper sizing using an Agilent Technologies 2100 bioanalyzer. The double stranded PCR products were heat denatured and circularized using a splint oligonucleotide that was homologous to the adaptor regions of the library to generate a single stranded cDNA circle that was closed with a ligation reaction. This was followed by an exonuclease digestion to remove any single-stranded non-circularized cDNA. The single stranded circular cDNA was the final format of the sequencing library and underwent final linear amplification with phi29 polymerase (Rolling Circle Amplification) to generate 300-500 copies of the library which folded into DNA nanoballs (DNB). The resulting DNB library was quantified by fluorometry prior to sequencer loading.

The DNB library was flowed across a patterned nanoarray with positive surface charged “active sites” that were sized to bind a single DNB. Paired-end 100 bp sequencing was performed using combinatorial Probe-Anchor Synthesis (cPAS), which involves initial hybridization of sequencing primers (anchors) to the adaptor region of the DNB followed by sequential incorporation of a fluorescently labeled dNTP, imaging of the flow cell, and removal of the fluorescent dye which enables continued sequencing by synthesis cycles. Data output for each sample was greater than 50,000,000 clean reads (adaptor and low-quality read trimmed).

Using the cloud-based virtual instance of the Galaxy bioinformatics analysis environment described in *Sequencing Data Analysis Platform* section, adaptor trimmed, clean reads (FASTQ) were mapped using RNA STAR(21), a gapped-read mapper for RNA sequencing data analysis using settings for paired-end reads and the mouse mm10 reference genome. The alignment data output (BAM) from RNA STAR was then loaded into featureCounts (20), a read summarization program that counts sequence reads to each gene in a supplied annotation file. Exons are defined as features, and genes are defined as meta-features, with counts being summarized at the meta-feature or gene level. Reads or fragments that overlap multiple genes are excluded from the counts. The featureCounts built-in annotation for the mouse genome, mm10, was used, and an output format producing a tabular file containing counted reads, per gene, per row, with a last column of effective gene-length. Finally, counts files for each sample were analyzed with edgeR(22, 23) to determine differentially expressed genes between groups, using the edgeR quasi-likelihood pipeline (edgeR-quasi) for differential expression analysis. This statistical methodology uses negative binomial generalized linear models, but with F-tests instead of likelihood ratio tests. This method provides stricter error rate control than other negative binomial based pipelines, including the traditional edgeR pipelines or DESeq2(24). While limma(25) pipelines are recommended for large-scale datasets, because of their speed and flexibility, the edgeR-quasi pipeline gives better performance in low-count situations including complex tissues such as adipose tissue fat pads. Final outputs from edgeR were a table of differentially expressed genes and a normalized counts table.

**References**
