# Supplementary material for: Identification of the transgene insertion site for an adipocyte-specific adiponectin-cre model and characterization of the functional consequences
Source: Adipocyte. 2021 Feb 10;10(1):91–100. doi: 10.1080/21623945.2021.1880083 (PMC7889145; doi:10.1080/21623945.2021.1880083)
Supplement: Supplemental Material [file KADI_A_1880083_SM8703.zip › Supplementary information/Supplemental.docx]

**Supplemental Table 1. Genotyping Primers**

**Supplemental Table 2. Primers for validation of Adipoq-cre transgene passenger gene expression and copy number**

Supplemental Figure 1. Informative mating scheme to determine whether the *Adipoq*-*Cre* transgene and the *Adam10* locus are linked. One founder (in this instance, the male) is hemizygous for the Adipoq-Cre transgene and heterozygous for the floxed allele at the Adam10 locus. The other founder (female) does not carry the Adipoq-Cre transgene and is homozygous for the floxed allele at the Adam10 locus. Below the parental genotypes, Punnett squares display predicted allele combinations and the combined probability of generating a progeny that is hemizygous for the Adipoq-Cre transgene and homozygous for the floxed allele at the Adam10 locus. In this breeding arrangement it is expected that 25% of progeny, on average, would have this combined genotype.

Supplemental Figure 2. Gene expression in non-adipose tissue. Gene expression in (A) liver, (B) heart, (C) brain, and (D) kidney all normalized to *Rpp30,* *Adipoq*-Cre^+/-^ (grey bars) and control littermates (open bars). E. Organ weight at euthanasia, *Adipoq*-Cre^+/-^ (grey bars) and control littermates (open bars). All values are mean + SEM (n=3/group); ***p*<0.01; *****p*<0.0001.

**Supplemental Figure 3. Gene expression in mouse tissue types.** (A) *Adipoq*, (B) *Eif4a2*, (C) *Kng1*, (D) *Kng2*, (E) *Rfc4*, (F) *Tbx18* gene expression in mouse tissue types. Mouse ENCODE transcriptome data, profiling developmental and mature tissues, demonstrating high and selective gene expression in the adult genital fat pad, subcutaneous fat pad, and mammary gland. RPKM, reads per kilobase per million reads placed.
